# Clinical significance of the m6A methyltransferase METTL3 in peripheral blood of patients with coronary heart disease

**DOI:** 10.3389/fcvm.2024.1442098

**Published:** 2024-09-20

**Authors:** Jianshe Chang, Rui Shao, Xiangshan Xu, Yuanzhe Jin

**Affiliations:** ^1^ Department of Cardiology, Fourth Affiliated Hospital of China Medical University, Shenyang, China; ^2^ Key Laboratory of Liaoning Provincial Medicine and Engineering of Cardiovascular Fluid Dynamics, China Medical University, Shenyang, China; ^3^ Department of Cardiology, North China Medical Treatment Health Group, Fengfeng General Hospital, Handan, China; ^4^ Department of Intensive Care, North China Medical Treatment Health Group, Fengfeng General Hospital, Handan, China

**Keywords:** coronary heart disease, m6A, METTL3, Gensini score, peripheral blood

## Abstract

**Objective:**

This study aims to explore the association of methyltransferase-like protein 3 (METTL3) expression with severity of coronary artery stenosis in patients with coronary heart disease (CHD).

**Methods:**

A total of 100 patients administrated in the Fourth Affiliated Hospital of China Medical University between October 2022 and June 2023 with primary symptoms of chest pain or tightness, or cardiac discomfort, and who underwent coronary angiography for a definitive diagnosis, were included in the study. The baseline characteristics, including TG, TC, LDL-C, HDL-C, uric acid and past history were recorded. Peripheral blood samples were collected to assess the expression levels of METTL3, YT521-B homology domains 1 (YTHDF1), YT521-B homology domains 2 (YTHDF2), and YT521-B homology domains 3 (YTHDF3) using the PCR method. Relative expression levels of METTL3 protein were determined by Western blotting. Correlation analysis were conducted to evaluate the relationship between METTL3/YTHDF1 gene expression and clinical data. Receiver operating characteristic (ROC) curve analysis was employed to assess the predictive value of METTL3 and YTHDF1 for CHD. Binary logistic regression was used to determine whether the expression of METTL3 and YTHDF1 in peripheral blood were risk factors for CHD.

**Results:**

The study found no significant differences in baseline characteristics between CHD patients and controls, except for length of stay, Lymphocytes, Neutrophils, AST, HDL-C and modified Gensini score. The gene expression levels of METTL3 and YTHDF1 were significantly higher in CHD patients compared to controls (*p* < 0.05). Furthermore, METTL3 protein expression was also significantly elevated in the CHD group compared to the control group (*p* < 0.05). METTL3 gene expression correlated with HDL-C and Gensini score, while YTHDF1 gene expression correlated with Age, WBC, Neutrophils, RDW-CV, modified Gensini score. ROC curve analysis demonstrated an AUC of 0.692 for METTL3 in CHD, with a sensitivity of 66.7% and a specificity of 69.8% at a cut-off value of >0.052. The AUC for YTHDF1 in CHD was 0.623, with a sensitivity of 47.4% and a specificity of 74.4% at a cut-off value of >0.027. Binary logistic regression revealed that only increased METTL3 expression in peripheral blood was an independent risk factor for CHD (*p* < 0.05).

**Conclusions:**

The increased expression of METTL3 in peripheral blood may serve as a potential biomarker and predictive factor for CHD.

## 1 Introduction

Coronary heart disease (CHD) is a prevalent cardiovascular disease associated with high morbidity and mortality, however, its pathogenesis is still not fully understood (1, 2). Therefore, exploring the molecular mechanisms underlying CHD pathogenesis is crucial for the development of effective diagnostic and therapeutic strategies (3). However, the current non-invasive methods used to diagnose CHD have low sensitivity and specificity, while coronary angiography, the gold standard for the diagnosis of CHD, is invasive (4). Consequently, the identification of specific biomarkers is essential for the early diagnosis of CHD (5).

N6-methyladenosine (m6A) modification is a common epigenetic modification in eukaryotes. The m6A modification is regulated by a group of enzymes, including the “writers” methyltransferase complex responsible for N6-adenylate methylation, the “erasers” demethyltransferases that remove N6-adenylate methylation, and the “readers” m6A RNA-binding proteins that are responsible for the biological function (6). Among them, METTL3 and methyltransferase-like 14 (METTL14) serve as key methyltransferases, fat mass and obesity-associated protein (FTO) and alkylation repair homolog protein 5 (ALKBH5) mainly function as demethyltransferases, while YTH protein family including YTHDF1, YTHDF2, and YTHDF3 are RNA-binding proteins which are capable of recognizing m6A modifications and control RNA fate (7). Studies have shown that abnormal expression of key m6A regulatory factors could be used as diagnostic and therapeutic targets in various diseases (7–9). For instance, Guo found a positive correlation between METTL14 levels and inflammatory markers, establishing it as an independent predictor of CHD risk (10). However, the correlation between the levels of m6A regulatory proteins in peripheral blood of patients with coronary artery stenosis severity remains unclear.

In this research, we measured the level of METTL3, YTHDF1, YTHDF2, and YTHDF3 in the peripheral blood of patients with CHD. The study revealed that the expression of METTL3 and YTHDF1 were increased in the CHD group. Furthermore, the expression of METTL3 and YTHDF1 was significantly correlated with various clinical parameters including HDL-C, Gensini score, Age, WBC, neutrophil, and RDW-CV. The Receiver Operating Characteristic (ROC) curve demonstrated that METTL3 had a strong predictive value in distinguishing the CHD group from the control group. Binary Logistic regression analysis indicated that increased METTL3 expression is an independent risk factor for CHD. These findings may provide new insights into new diagnostic biomarker for CHD.

## 2 Materials and methods

### 2.1 Patients enrollment

A total of 100 consecutive patients who administrated in the Cardiology department of 4th Affiliated Hospital of China Medical University between October 2022 and June 2023 with primary symptoms of chest pain or tightness, or cardiac discomfort, and who underwent coronary angiography for a definitive diagnosis, were included in this study. This study was approved by the hospital Ethics Committee, and all participants provided written informed consent.

Based on the results of coronary angiography, the patients were categorized into CHD group and control group. The inclusion criteria for the CHD group included the presence of more than 50% stenosis in at least one of the coronary arteries (left main, left circumflex, left anterior descending, or right coronary artery). The control group consisted of patients with no more than 50% coronary artery stenosis or only myocardial bridge changes. Patients who had undergone coronary stent implantation and coronary artery bypass grafting were excluded, as well as those with hematological diseases, tumors, drug abuse, pregnant, precancerous lesions, severe hepatic and renal insufficiency, active bleeding of various causes, and chronic obstructive pulmonary disease.

### 2.2 Peripheral blood samples collection and double-stranded DNA (dsDNA) extraction

In this study, trained staff collected all subject laboratory indicators. 6 ml of peripheral venous blood was obtained from the patient prior to coronary angiography. 5 ml of the blood sample was sent to the hospital laboratory for blood routine test, blood glucose, blood lipids, uric acid, and other indicators. The remaining 1 ml of venous blood was collected in an anticoagulant tube containing EDTA for dsDNA extraction. The extraction of Peripheral Blood dsDNA was carried out using the FastPure® Blood DNA Isolation Mini Kit V2 (Vazyme, Nanjing, China) following the manufacturer's protocol. The concentration and purity of the dsDNA were assessed using a NanoDrop spectrophotometer (Thermo Fisher Scientific, Waltham, USA).

### 2.3 Polymerase chain reaction (PCR)

PCR was conducted using the Applied Biosystems QuantStudio 3 (Thermo Fisher Scientific, USA) using ChamQ Universal SYBR qPCR master mix (Vazyme, Q711-02). The primers for METTL3, YTHDF1, YTHDF2, YTHDF3, and GAPDH can be found in Table 1. All PCR experiments were conducted in triplicate. The relative expression of the aforementioned genes was calculated using the 2^−ΔCt^ method (11).

**Table 1:** The PCR sequences.

Gene	Sequence (F:5′–3′)	Sequence (R:5′–3′)
GAPDH	CATACCAGGAAATGAGCTTG	ATGACATCAAGAAGGTGGTG
METTL3	AGATGGGGTAGAAAGCCTCCT	TGGTCAGCATAGGTTACAAGAGT
YTHDF1	GCCTGAAGATTGGGGACG	GGTCGGCTTTGAAACTGG
YTHDF2	TTTCAGTCCAGCAACAGG	ATTACCATCCACCCCATT
YTHDF3	ATGCGTATGCTGGTGTCT	CCTCTTGAGTGTCCCTTGA

### 2.4 Protein extraction from whole blood and western blot (WB) analysis

Whole blood proteins were extracted from the collected blood samples using the Solarbio Whole Blood Protein Extraction Kit, following the manufacturer's instructions. An appropriate volume of whole blood was mixed with the provided lysis buffer to ensure complete cell lysis. The mixture was then incubated on ice for 30 min to facilitate protein solubilization. After centrifugation at 14,000 × g for 10 min at 4°C, the supernatant containing the extracted proteins was collected and stored at −80°C for further analysis.

For Western blotting, the extracted whole blood proteins were quantified using the BCA protein assay. Equal amounts of protein (typically 30 µg) were loaded onto a 10% SDS-PAGE gel and separated by electrophoresis. The proteins were subsequently transferred to a PVDF membrane. The membrane was incubated overnight at 4°C with a primary antibody against METTL3 (Cell Signaling Technology, USA), diluted at a ratio of 1:1,000. After washing, the membrane was incubated with an appropriate secondary antibody for 1 h, followed by additional washing steps. Finally, protein bands were detected using enhanced chemiluminescence (ECL), quantified with ImageJ software, and assembled into figures using Adobe Photoshop 2022. Statistical analysis and graphing were performed using GraphPad Prism 8.0.2.

### 2.5 Modified Gensini score calculation

According to the results of quantitative coronary angiography, we utilized the modified Gensini Score to assess the severity of coronary artery. The modified Gensini Score assigns different scores based on the degree of stenosis of the coronary artery (1 for 0%–25% stenosis, 2 for 26%–50%, 4 for 51%–75%, 8 for 76%–90%, 16 for 91%–99%, and 32 for 100%). If the lesion was ≥99%, the score was adjusted based on the presence of collateral supplying arteries and the degree of narrowing of the supplying arteries. Additionally, different multiplier factors were assigned based on the specific positions of the coronary artery tree where the lesions were located (5 for the left main coronary artery, 2.5 for the proximal left anterior descending branch and the proximal left circumflex branch, 1.5 for the midsegment of left anterior descending artery, 0.5 for the second diagonal branch and the posterolateral branch, and 1 for other branches) (12, 13). The modified Gensini score for each enrolled patient was the total of all lesion severity scores. The higher the score is, the more severe the coronary is.

### 2.6 Statistical analysis

All statistical analyses were performed using GraphPad Prism v8.0 software (GraphPad Software) and SPSS version 26.0 software (SPSS Inc.). For measurement data, we initially utilized the Kolmogorov-Smirnov test to assess the data distribution. Data that followed a normal distribution were presented as mean ± standard deviation, and an independent sample *T*-test was used to compare the two groups of data. Data that did not follow the normal distribution were presented as the median (interquartile range), and the Mann–Whitney *U* rank sum test was conducted for both groups. For counting data, expressed as [*n* (%)], the chi-square test was used for comparison between the two groups. Pearson or Spearman correlation analysis was utilized to assess the correlation between variables. ROC curves were performed to evaluate the diagnostic significance of METTL3 and YTHDF1. Binary Logistic regression analysis was employed to assess the risk factor. A *P* value < 0.05 was considered statistically significant.

## 3 Results

### 3.1 The baseline characteristics comparison between the two groups

This study included a total of 100 patients, 57 patients with CHD and 43 controls. The baseline characteristics of the two groups are outlined in Table 2. No significant differences were found between the two groups in terms of age, sex, history of hypertension, history of diabetes, smoking, WBC, PLT, RBC, HGB, RDW-CV, ALT, TBIL, DBIL, IBIL, TC, TG, LDL-C, UREA, Cr, UA, LVEDV, LVESV, LVSV, LVEF. However, there were statistically significant differences between the two groups in length of hospital stay, Lymphocytes, Neutrophils, AST, HDL-C and Gensini score.

**Table 2:** Baseline characteristics of patients in the two groups.

	CHD (*n* = 57)	Control (*n* = 43)	*p* value
Sex (Male/Female)	40/17	25/18	0.212
Age (year)	63.56 ± 8.28	60.16 ± 10.24	0.070
History of hypertension [*n* (%)]	37 (64.9%)	28 (65.1%)	0.983
History of diabetes [*n* (%)]	21 (36.8%)	9 (20.9%)	0.086
Smoking [*n* (%)]	22 (38.6%)	14 (32.6%)	0.533
Length of stay (day)	8 (7–10)	7 (5–8)	0.016*
WBC (10^9^/L)	7.4 (6.09–9.51)	6.8 (5.97–8)	0.159
Lymphocytes (10^9^/L)	1.53 (1.15–2.01)	1.76 (1.54–2.3)	0.021*
Neutrophils (10^9^/L)	5.15 (4.38–6.61)	4.31 (3.49–5.32)	0.009*
PLT (10^9^/L)	206 (176–256.5)	212 (191–244)	0.427
RBC (10^12^/L)	4.57 ± 0.44	4.67 ± 0.45	0.304
HGB (g/L)	137.51 ± 14.02	141.14 ± 14.41	0.208
RDW-CV (%)	12.62 ± 0.84	12.43 ± 0.86	0.271
GLU (mmol/L)	6.27 (5.4–8.89)	5.7 (4.99–5.38)	0.015*
ALT (U/L)	27 (18–40)	21 (16–35)	0.103
AST (U/L)	28 (20.5–60.5)	20 (18–29)	0.002*
TBIL (μmol/L)	12.05 (9.38–16.85)	12.2 (9.1–16)	0.534
DBIL (μmol/L)	4.1 (3–5.48)	3.3 (2.7–5)	0.123
IBIL (μmol/L)	8.6 (6.45–10.85)	8.1 (6.5–10.2)	0.818
TC (mmol/L)	4.32 ± 1.17	4.46 ± 1.15	0.544
TG (mmol/L)	1.6 (1.06–2.07)	1.37 (0.93–2.4)	0.857
HDL-C (mmol/L)	0.88 (0.76–1.02)	1.09 (0.87–1.26)	0.001*
LDL-C (mmol/L)	2.88 ± 1.13	2.73 ± 1.12	0.517
UREA (mmol/L)	5.5 (4.75–6.8)	5.83 (5.01–6.78)	0.503
Cr (μmol/L)	65 (52.5–75)	66.5 (55.8–71.5)	0.846
UA (μmol/L)	334 (263–385)	337.5 (281–382.8)	0.591
LVEDV (ml)	88 (74.5–112.3)	87 (65.5–100)	0.310
LVESV (ml)	34.5 (29–43)	32 (27–39)	0.247
LVSV (ml)	54 (45–65.8)	50 (41–60)	0.167
LVEF (%)	61 (58–64)	61 (60–64)	0.506
Gensini score	38 (20.75–45.5)	4 (2.5–7)	<0.001*

WBC, white blood cell count; PLT, platelet count; RBC, red blood cell count; HGB, hemoglobin; RDW-CV, red blood cell volume distribution width; GLU, glucose; ALT, alanine aminotransferase; AST, aspartate aminotransferase; TBIL, total bilirubin; DBIL, direct bilirubin; IBIL, indirect bilirubin; TC, total cholesterol; TG, triglyceride; HDL-C, high-density lipoprotein cholesterol; LDL-C, low-density lipoprotein cholesterol; UREA, urea; Cr, creatinine; UA, uric acid; LVEDV, left ventricular end-diastolic volume; LVESV, left ventricular end-systolic volume; LVSV, left Ventricular stroke volume; LVEF, left ventricular ejection fraction.

*Values denote significance level at *p* < 0.05.

### 3.2 Differential expression levels of METTL3, YTHDF1, YTHDF2, and YTHDF3 genes and METTL3 protein in peripheral blood of CHD patients compared to controls

**Figure 1:**
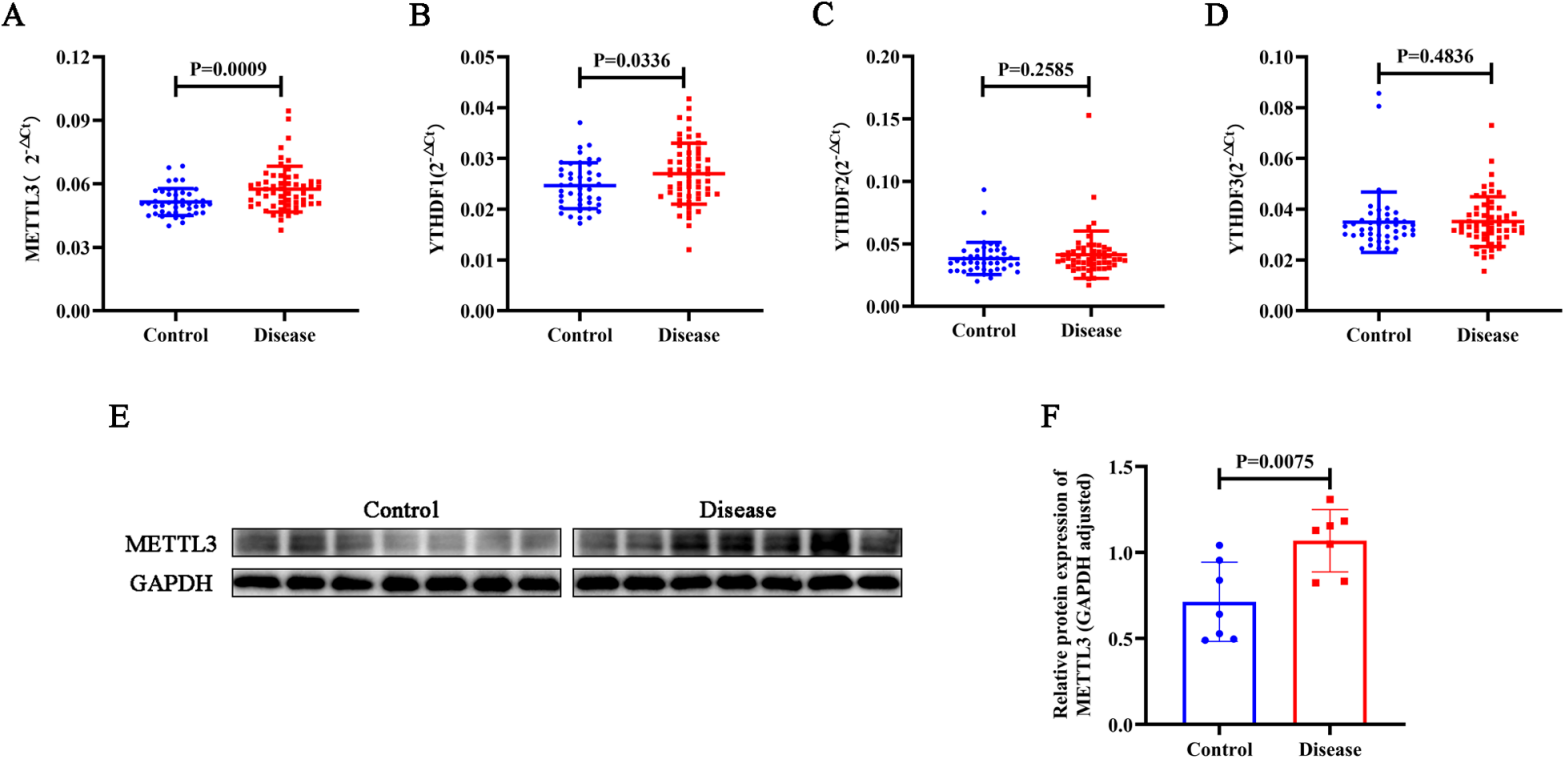
Using PCR, the gene expression levels of METTL3 **(A)**, YTHDF1 **(B)**, YTHDF2 **(C)**, and YTHDF3 **(D)** were assessed in peripheral blood samples from CHD patients and controls. **(E–F)** The protein expression levels of METTL3 were assessed via WB analysis in CHD patients and controls, with GAPDH protein serving as an internal control.

To compare the levels of METTL3, YTHDF1, YTHDF2, and YTHDF3 in patients with CHD and the control group, peripheral blood samples were collected for expression analysis using PCR. As depicted in Figures 1A–D, the gene expression levels of METTL3 and YTHDF1 in the peripheral blood of CHD patients were significantly higher than those in the control group (*p* < 0.05), whereas the levels of YTHDF2 and YTHDF3 did not differ significantly (*p* > 0.05). Subsequently, we conducted a WB experiment on the blood samples. As shown in Figures 1E,F, the expression of METTL3 protein was significantly elevated in the CHD group compared to the control group (*p* < 0.05).

### 3.3 Correlation between METTL3 and YTHDF1 in peripheral blood and clinical data in all patients

**Figure 2:**
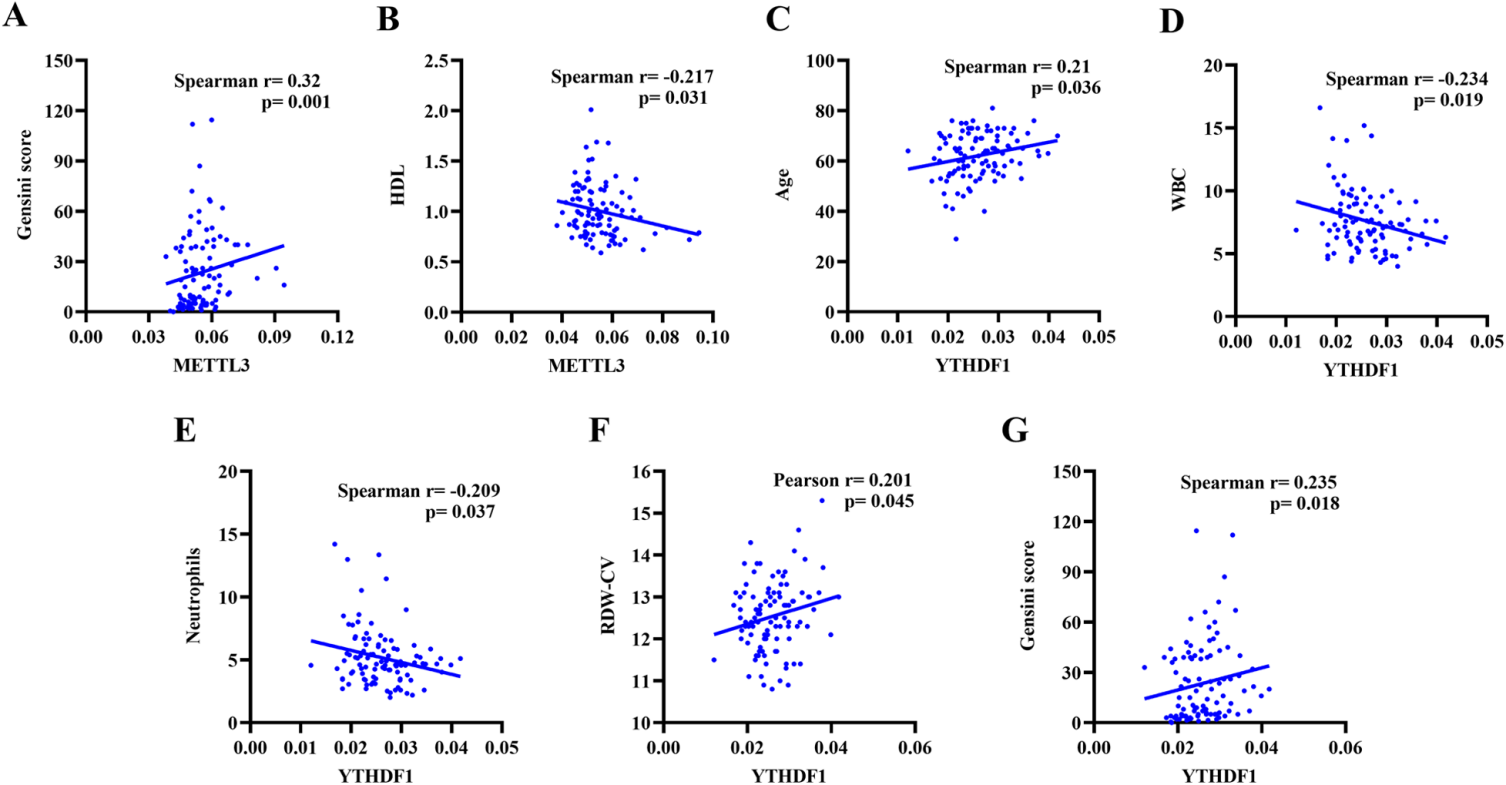
Correlation analysis of METTL3 gene expression level with Gensini score **(A)**, HDL **(B)**, and Age **(C)**. Correlation analysis of YTHDF1 gene expression level with WBC **(D)**, Neutrophils **(E)**, RDW-CV **(F)**, and Gensini score **(G)**.

The correlation analysis was conducted to assess the relationship between the clinical data (Age, Length of stay, WBC, Lymphocytes, Neutrophils, PLT, RBC, HGB, RDW-CV, ALT, TBIL, DBIL, IBIL, TC, TG, HDL-C, LDL-C, UREA, Cr, UA, LVEDV, LVESV, LVSV, LVEF, Gensini score) and the expression of peripheral blood METTL3 and YTHDF1. As shown in Table 3 and Figure 2, it demonstrated that the expression of METTL3 in peripheral blood displayed negative correlation with HDL-C (*r* = −0.217, *p* = 0.031) and positive correlation with modified Gensini score (*r* = 0.320, *p* = 0.001). Additionally, the expression of YTHDF1 in peripheral blood was significant associated with Age (*r* = 0.210, *p* = 0.036), WBC (*r* = −0.234, *p* = 0.019), Neutrophils (*r* = −0.209, *p* = 0.037), RDW-CV (*r* = 0.201, *p* = 0.045), Gensini score (*r* = 0.235, *p* = 0.018). However, there was no correlation between the expression of METTL3 and YTHDF1 with other clinical data.

**Table 3:** Correlation between METTL3 and YTHDF1 in peripheral blood and clinical baseline characteristics.

	METTL3		YTHDF1	
	*r*	*p*	*r*	*p*
Age	0.133	0.186	0.210	0.036*
Length of stay	0.050	0.618	0.076	0.452
WBC	0.061	0.550	−0.234	0.019*
Lymphocytes	−0.102	0.312	−0.126	0.212
Neutrophils	0.101	0.315	−0.209	0.037*
PLT	−0.114	0.260	−0.091	0.369
RBC	0.088	0.382	−0.012	0.903
HGB	0.046	0.649	0.033	0.743
RDW-CV	−0.005	0.959	0.201	0.045*
GLU	0.114	0.261	0.025	0.806
ALT	0.150	0.141	0.033	0.751
AST	0.127	0.208	−0.072	0.477
TBIL	0.029	0.777	0.033	0.744
DBIL	0.064	0.531	0.047	0.644
IBIL	−0.001	0.991	−0.009	0.931
TC	0.062	0.543	0.063	0.535
TG	0.062	0.541	−0.082	0.418
HDL-C	−0.217	0.031*	−0.050	0.624
LDL-C	0.035	0.731	0.028	0.780
UREA	0.038	0.709	0.051	0.613
Cr	0.124	0.223	0.106	0.298
UA	0.123	0.226	0.094	0.355
LVEDV	0.136	0.187	0.135	0.193
LVESV	0.085	0.415	0.149	0.149
LVSV	0.184	0.074	0.084	0.416
LVEF	0.112	0.281	−0.108	0.297
Gensini score	0.320	0.001*	0.235	0.018*

WBC, white blood cell count; PLT, platelet count; RBC, red blood cell count; HGB, hemoglobin; RDW-CV, red blood cell volume distribution width; GLU, glucose; ALT, alanine aminotransferase; AST, aspartate aminotransferase; TBIL, total bilirubin; DBIL, direct bilirubin; IBIL, indirect bilirubin; TC, total cholesterol; TG, triglyceride; HDL-C, high-density lipoprotein cholesterol; LDL-C, low-density lipoprotein cholesterol; UREA, urea; Cr, creatinine; UA, uric acid; LVEDV, left ventricular end-diastolic volume; LVESV, left ventricular end-systolic volume; LVSV, left ventricular stroke volume; LVEF, left ventricular ejection fraction.

*Values denote significance level at *p* < 0.05.

### 3.4 Diagnostic predictive value of METTL3 and YTHDF1 for CHD

**Figure 3:**
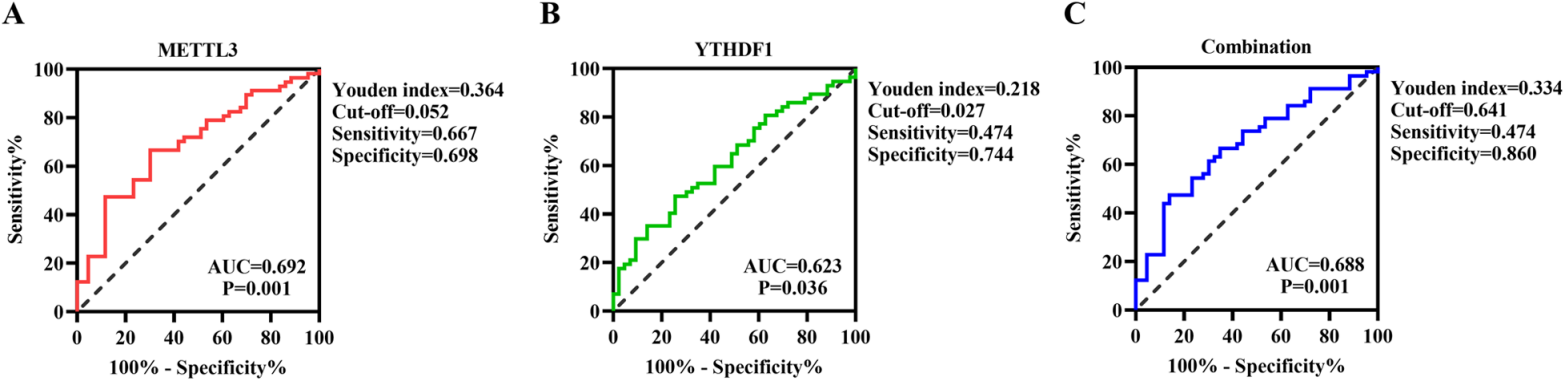
ROC curve analysis of METTL3 **(A)**, YTHDF1 **(B)**, and their combination **(C)**.

Subsequently, we conducted a ROC curve analysis to investigate the potential predictive value of METTL3 and YTHDF1 in peripheral blood for CHD. The results showed that the AUC of METTL3 for distinguishing CHD from Control was 0.692, with a cutoff value of >0.052, a sensitivity of 66.7%, and a specificity of 69.8% (Figure 3A). The AUC of YTHDF1 was 0.623, with a cutoff value of >0.027, a sensitivity of 47.4%, and a specificity of 74.4% (Figure 3B). Furthermore, the AUC of the combination of METTL3 and YTHDF1 for distinguishing CHD from Control was 0.688, with a sensitivity of 47.4% and a specificity of 86% (Figure 3C). It is noteworthy that the combination of METTL3 and YTHDF1 (0.688) did not show improvement in predicting patients with CHD compared to the sole use of METTL3 (0.692).

### 3.5 The increased expression of METTL3 was a risk factor for CHD

The previous results revealed that the increased expression of METTL3 and YTHDF1 in peripheral blood were associated with severity of coronary artery. To further investigate the association between the expression of METTL3 and YTHDF1 in peripheral blood and the risk of CHD, binary logistic regression was employed. The results, as shown in Table 4, yielded the equation *Y* = 0.282 + 0.088 X1(METTL3*1000) + 0.007 X2 (YTHDF1*1000), indicating that only the increased expression of METTL3 in peripheral blood was an independent risk factor for CHD (*p* < 0.05).

**Table 4:** The expression of METTL3 and YTHDF1 in equation.

	B	SE	Wald	df	Exp (B)	*p*
METTL3	0.088	0.036	5.857	1	1.092	0.016*
YTHDF1	0.007	0.050	0.020	1	1.007	0.888
Constant	0.282	0.202	1.947	1	1.326	0.163

*Values denote significance level at *p* < 0.05.

## 4 Discussion

M6A modification is a common and important type of RNA modification regulated by m6A methylation regulators (14). Dysregulation of RNA m6A modification is increasingly recognized as a contributing factor in many diseases, including cardiovascular diseases (15–17). Our study found that the expression levels of the METTL3 gene and protein in peripheral blood were significantly increased in patients with CHD, and this expression was positively associated with the severity of coronary artery disease. Furthermore, this paper revealed that METTL3 is an independent risk factor for CHD, providing valuable insights into its potential as a biomarker and risk factor.

Patients in the CHD group showed increased expression of METTL3 and YTHDF1 in peripheral blood, indicating a potential association between m6A modification and CHD. Li et al. found that the expression of METTL3 in macrophages increased with the progression of atherosclerosis and METTL3 targets BRAF mRNA and promoted its translation through YTHDF1, exacerbating macrophage-mediated inflammatory responses and atherosclerotic progression (18). Additionally, Chien et al. found that METTL3 in endothelial cells mediated inflammatory responses under pro-atherosclerotic flow treatment, including secretion of inflammatory cytokines, activation of the NF-*κ*B signaling pathway, and adhesion of monocytes to endothelial cells, exacerbating the development of atherosclerosis (19). On the other hand, Li et al. found that METTL3 mitigates the progression of atherosclerosis by regulating m6A-dependent EGFR mRNA stability (20). While the roles of METTL3 in the above results are not entirely consistent, all findings indicate a connection between dysregulated m6A modification and the pathogenesis of CHD.

In this study, we explored the associations between the expression of METTL3 and YTHDF1 and clinical parameters. Specifically, we observed a positive correlation between the level of METTL3 and modified Gensini score, and a negative correlation with HDL-C levels. These findings imply that METTL3 could potentially serve as a risk factor for CHD. Additionally, YTHDF1 expression was positively correlated with age, RDW-CV, and modified Gensini score, and negatively correlated with WBC and Neutrophil levels. These correlations highlight the complex interactions between m6A modification regulatory molecules and various clinical parameters, providing evidence for the potential impact of RNA m6A modification on the pathological and physiological processes of CHD.

The ROC curve analysis demonstrated the potential predictive value of METTL3 and YTHDF1 for CHD, with the AUC for METTL3 being 0.692 and for YTHDF1 being 0.688. However, the combination of METTL3 and YTHDF1 did not significantly improve predictive capability compared to the use of METTL3 alone. These findings underscore the potential utility of METTL3 as a biomarker for CHD. Building on these results, we further evaluated the expression levels of METTL3 protein in blood samples through WB analysis. The results showed that METTL3 protein expression levels were significantly elevated in the CHD group compared to the control group. Moreover, the binary logistic regression analysis revealed that only the increased expression of METTL3 in peripheral blood was identified as an independent risk factor for CHD. This observation highlights the potential significance of METTL3 as a risk factor for CHD and suggests its potential ability in risk stratification and prognostication for patients with CHD.

Although our study has produced some findings, it also has limitations that need to be acknowledged. Firstly, the research is based on a small sample from a single center, and some important information in the medical records—such as family history of premature CHD, inflammatory factors (CRP, IL-6, etc.), homocysteine levels, and drug therapy information—is incomplete. Secondly, our research may be subject to certain information biases, such as selection bias. Additionally, external factors, such as variations in treatment regimens and differences in patient populations, may have influenced our study. Therefore, in the future, it is necessary to validate the diagnostic and prognostic utility of METTL3 and YTHDF1 in larger samples and to explore their roles in the pathogenesis of CHD.

In conclusion, this study found that the expression level of METTL3 in peripheral blood is significantly increased in CHD patients and its expression level is positive associated with the severity of coronary artery. Furthermore, METTL3 is an independent risk factor of CHD, which providing valuable insights into its potential as a biomarker and risk factor for CHD.

## Data Availability

The raw data supporting the conclusions of this article will be made available by the authors, without undue reservation.
